# Effect of the Nipple-Excising Breast-Conserving Therapy in Female Breast Cancer: A Competing Risk Analysis and Propensity Score Matching Analysis of Results Based on the SEER Database

**DOI:** 10.3389/fonc.2022.848187

**Published:** 2022-04-14

**Authors:** Shouyu Li, Yuting Zhao, Lutong Yan, Zejian Yang, Pei Qiu, Heyan Chen, Yudong Zhou, Ligang Niu, Yu Yan, Wei Zhang, Huimin Zhang, Jianjun He, Can Zhou

**Affiliations:** ^1^ Department of Breast Surgery, First Affiliated Hospital of Xi’an Jiaotong University, Xi’an, China; ^2^ Xi’an Jiaotong University Health Science Center, Xi’an, China

**Keywords:** nipple areola complex, female breast cancer, breast-conserving therapy, SEER database, competing risk model

## Abstract

**Introduction:**

Due to the lack of randomized controlled trial, the effectiveness and oncological safety of nipple-excising breast-conserving therapy (NE-BCT) for female breast cancer (FBC) remains unclear. We aimed to explore and investigate the prognostic value of NE-BCT versus nipple-sparing breast-conserving therapy (NS-BCT) for patients with early FBC.

**Methods:**

In this cohort study, data between NE-BCT and NS-BCT groups of 276,661 patients diagnosed with tumor–node–metastasis (TNM) stage 0–III FBC from 1998 to 2015 were retrieved from the Surveillance, Epidemiology, and End Results database. Propensity score matching analysis, Kaplan–Meier, X-tile, Cox proportional hazards model, and competing risk model were performed to evaluate the effectiveness and oncological safety for patients in NE-BCT and NS-BCT groups.

**Results:**

A total of 1,731 (0.63%) patients received NE-BCT (NE-BCT group) and 274,930 (99.37%) patients received NS-BCT (NS-BCT group); 44,070 subjects died after a median follow-up time of 77 months (ranging from 1 to 227 months). In the propensity score matching (PSM) cohort, NE-BCT was found to be an adversely independent prognostic factor affecting overall survival (OS) [hazard ratio (HR), 1.24; 95% CI, 1.06–1.45, *p*=0.0078]. Subjects in NE-BCT group had similar breast-cancer-specific survival (BCSS) (HR, 1.15; 95%CI, 0.88–1.52, *p*=0.30) and worse other-causes-specific death (OCSD) (HR, 1.217; 95%CI, 1.002–1.478, *p*=0.048<0.05) in comparison with those in the NS-BCT group.

**Conclusions:**

Our study demonstrated that the administration of NE-BCT is oncologically safe and reliable and can be widely recommended in clinics for women with non-metastatic breast cancer.

## Introduction

Due to the improving understanding of the biology of female breast cancer (FBC) and the advancement of radiotherapy and anti-cancer drugs over the past four decades, the proportion of breast-conserving therapy (BCT) is increasing in clinical works. Meanwhile, nipple-sparing breast conservation therapy (NS-BCT) followed by whole breast irradiation has been the standard therapy regimen by the National Comprehensive Cancer Network (NCCN) guidelines and European Society for Medical Oncology Clinical Practice Guidelines for patients with early breast cancer (1, 2). This procedure involves preservation of the nipple areolar complex (NAC) and breast when performing BCT in an attempt to get improved aesthetic outcomes and satisfaction and enhanced psychosocial and sexual wellbeing without reducing oncological safety in comparison with non-conservative mastectomy (3, 4) and has thus become increasingly attractive to both patients and doctors (5–7). However, the value of NAC remains controversial on account of the concern of possible residual cancer in the nipple (8).

Negative margins, defined as no invasive carcinoma on ink in the margins or obtaining margins >2 mm for patients with pure ductal carcinoma *in situ* (DCIS), are the standard procedure for BCT in patients with invasive breast carcinoma (1). The preservation of NAC can only be performed for patients with no evidence of cancer cell at the margin of the nipple. Thus, the procedure of NS-BCT is inappropriate for all subjects who are willing to receive BCT in the actually clinical work. Some patients are at considerably greater risk of excising NAC due to the nipple involvement, defined as DCIS, invasive carcinoma, or Paget’s disease within 1 cm of the NAC (9). The procedure of nipple-excising breast conserving therapy (NE-BCT) is passively taken to be performed for FBC patients with NAC involvement. The excision of NAC results in low satisfaction and psychosocial and sexual wellbeing in comparison with the preservation of NAC (7, 10). Nevertheless, most studies have focused on the effects of NS-BCT or nipple-sparing mastectomy on long-term outcomes for patients with FBC (11–16). The effectiveness and safety of NE-BCT on the long-term survival is very limited, and its benefits remain unclear, as there is no study investigating the oncological safety and clinical efficacy of NE-BCT. Therefore, it is necessary to explore and investigate the clinical issues and value of NE-BCT.

To further explore and identify the long-term oncological safety and clinical effects of NE-BCT in patients with FBC, we followed a large cohort of women who received BCT from 1998 to 2015 based on the population-based database Surveillance, Epidemiology, and End Results (SEER) cancer registry program. Statistical methods such as Cox proportional hazards model and competing risk analysis model were performed to further investigate the efficiency of NE-BCT and prognostic factors on patients with non-metastatic FBC.

## Methods

### Data Source

The data from the National Cancer Institute’s SEER program database (https://seer.cancer.gov/seerstat) (Information Management Service, Inc., Calverton, MD, USA), maintained by the National Cancer Institute, cover about one-third of the US population and are used in the current study. Subjects with FBC are abstracted from the SEER data by the SEER*Stat version 8.3.6.1 (SEER ID: 13148-Nov2019) in May 2020. All procedures were carried out in accordance with the approved guidelines. As the SEER database was publicly accessible to all people worldwide before January 2021, informed patient consent was not required for this research. Moreover, the study was approved by the Ethics Committee of the First Affiliated Hospital of Xi’an Jiaotong University.

### Cohort Selection

**Figure 1:**
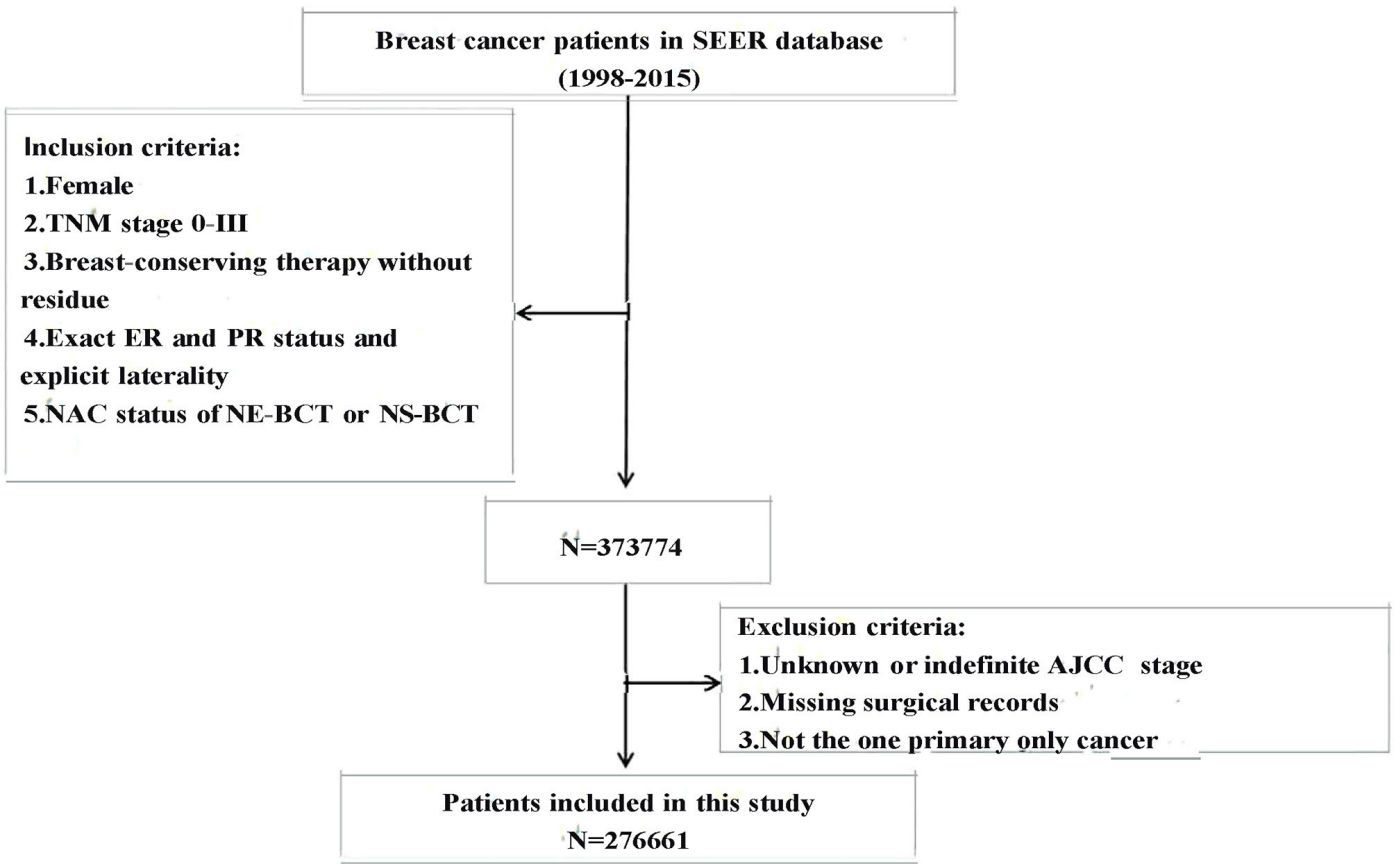
Inclusion and exclusion criteria of the study population.

Participants diagnosed with FBC and who underwent BCT between January 1, 1998 and December 31, 2015 were selected according to the following criteria: women (1) who were diagnosed as TNM [breast-adjusted American Joint Committee on Cancer (AJCC) 6th] stages 0, I, II, or III; (2) who underwent BCT without residual tumor; (3) with known ER and PR status; and (4) who underwent NE-BCT or NS-BCT. Patients conforming with the following criteria were excluded: (1) unknown or indefinite AJCC stage, (2) missing surgical records, and (3) not one primary cancer. The selection procedure is shown in **Figure 1**.

The following data were collected for each patient in the current study: age at diagnosis, race, year of diagnosis, marital status, laterality, tumor differentiation grade, ER status, PR status, radiotherapy status, chemotherapy status, surgical procedures, survival months, causes of death from the SEER database, and breast-adjusted AJCC 6th.

In total, 276,661 candidates who underwent BCT were selected. “No radiation and/or cancer-directed surgery” was considered as no radiotherapy. “No/unknown” chemotherapy recodes were considered as no chemotherapy. The administration of BCT contained segmental mastectomy, resections of biopsy sites for gross or microscopic residual diseases, tumor resection or biopsy, and partial mastectomy with papillectomy.

### Outcomes

All the patients enrolled were effectively followed up; the final follow-up time was November 2018. The primary end point was breast cancer-specific survival (BCSS), defined as the time from the beginning of diagnosis of FBC to the survival caused by breast cancer. The secondary outcome measurements were overall survival (OS), breast cancer-specific death (BCSD), and other-causes-specific death (OCSD). OS was defined as the time from the date of diagnosis to death; BCSD and OCSD were defined as the time from the date of diagnosis to the date of death from breast cancer and the other causes, respectively.

### Covariates

Covariates included the clinicopathological characteristics, such as age at diagnosis, race and origin recode, year of diagnosis, marital status, laterality of tumor, histological grade, ER status, PR status, HER2 status, T stage, N stage, surgical procedures, radiation status, and chemotherapy status.

### Statistical Analysis

The Pearson chi-square test or Fisher’s exact test was administrated to test the difference in patients’ clinicopathological characteristics among groups. Categorical variables were reported as the number of cases and percentages. PSM was performed to balance the differences in clinicopathological factors used between the two groups by 1:1 ratio matching. The PSM program and standardization difference were calculated by the nearest-neighbor matching method with a caliper distance of 0.05 and R packages of “MatchIt” (17). Then, the resulting score-matched pairs were performed in subsequent analyses.

Kaplan–Meier curve analysis was employed to generate OS and BCSS curves, with the log-rank test performed to determine the statistical differences among groups through R package of “survival” and “survminer.” Multivariate Cox regression analysis was used to explore the prognostic factors independently associated with OS in our research by R package of “survival” and “survminer.”

A competing risk model analysis was implied to mitigate the estimation bias by classifying the cause of deaths into BCSD and OCSD subgroups. In the multivariate survival competing risk analysis, the Fine and Gray competitive risk regression was adopted to identify factors related to risk of death from all causes, with the purpose of decreasing the bias due to informative censoring by R package of “cmprsk” (18). All statistical analyses were carried out using IBM SPSS 22.0 software (IBM Corporation) and R statistical software version 4.0.3. All statistical tests were two-sided, and the level of significance was set at *p* < 0.05.

## Results

### Patients Descriptive Characteristics

Among the 276,661 candidates originally identified in our study, 1,731 (0.63%) patients received NE-BCT (NE-BCT group), while 274,930 (99.37%) patients received NS-BCT (NS-BCT group). By comparing the clinicopathological characteristics between NE-BCT and NS-BCT groups, significant differences (*p*<0.05) were found in most variables. PSM was employed to avoid the potential prognostic confounders that could affect the accuracy of the results. In the PSM cohort, a total of 3,460 subjects were registered in the whole cohort, with 1,730 patients in the NE-BCT group and 1,730 ones in the NS-BCT group, respectively. Insignificant differences were found in all key methodological characteristics between NE-BCT and NS-BCT groups (**Table 1**).

**Table 1:** Patient clinical and pathological characteristics.

Characteristics	All patients	NE-BCT	NS-BCT	χ2/F	p-value
	N=3,460	N=1,730	N=1,730		
Age at diagnosis(median)	62.21 ± 13.64	62.41 ± 13.60	62.02 ± 13.64	0.708	>0.05
Age				0.11	0.92
<65	1,935 (55.9)	969 (56.0)	966 (55.8)		
≧65	1,525 (44.1)	761 (44.0)	764 (44.2)		
Race				0.112	0.946
Non-Hispanic White	2,310 (66.8)	1,159 (67.0)	1,151 (66.5)		
Non-Hispanic Black	343 (9.9)	169 (9.8)	174 (10.1)		
Others	807 (23.3)	402 (23.2)	405 (23.4)		
Year of diagnosis				0.131	0.717
1998–2006	805 (23.3)	398 (23.0)	407 (23.5)		
2007–2015	2,655 (76.7)	1,332 (77.0)	1,323 (76.5)		
Marital status				0.07	0.79
Married	1,790 (51.7)	899 (52.0)	891 (51.5)		
Others	1,670 (48.3)	831 (48.0)	839 (48.5)		
Laterality				0.14	0.71
Left	1,819 (52.6)	915 (52.9)	904 (52.3)		
Right	1,641 (47.4)	815 (47.1)	826 (47.8)		
Grade				0.057	0.972
I	762 (22.0)	379 (21.9)	383 (22.1)		
II	1,640 (47.4)	819 (47.3)	821 (47.5)		
III+IV	1,058 (30.6)	532 (30.7)	526 (30.4)		
ER Status				0.002	0.96
Positive	2,875 (83.1)	1,437 (83.1)	1,438 (83.1)		
Negative	585 (16.9)	293 (16.9)	292 (16.9)		
PR Status				0.01	0.91
Positive	2,537 (73.3)	1,267 (73.2)	1,270 (73.4)		
Negative	923 (26.7)	463 (26.8)	460 (26.6)		
T Stage				0.76	0.69
T0–1	2,219 (64.1)	1,111 (64.2)	1,108 (64.1)		
T2	1,073 (31.0)	530 (30.6)	543 (31.4)		
T3–4	168 (4.9)	89 (5.1)	79 (4.6)		
N Stage				0.41	0.81
N0	2,441 (70.6)	1,229 (71.0)	1,212 (70.1)		
N1	827 (23.9)	406 (23.5)	421 (24.3)		
N2–3	192 (5.6)	95 (5.5)	97 (5.6)		
Radiation				0.11	0.74
Yes	2,335 (67.5)	1,172 (67.8)	1,163 (67.2)		
No	1,125 (32.5)	558 (32.3)	567 (32.8)		
Chemotherapy				0.08	0.78
Yes	1,190 (34.4)	591(34.2)	599 (34.6)		
No	2,270 (65.6)	1,139 (65.8)	1,131 (65.4)		

NE-BCT, nipple-excising breast-conserving therapy; NS-BCT, nipple-sparing breast-conserving therapy; ER, estrogen receptor; PR, progesterone receptor; HER-2, human epidermal growth factor receptor 2; BCSD, breast-cancer-specific death; OCSD, other-cause-specific death.

### BCSS and OS Curve Associated With NE-BCT vs. NS-BCT by Kaplan–Meier Analysis

**Figure 2:**
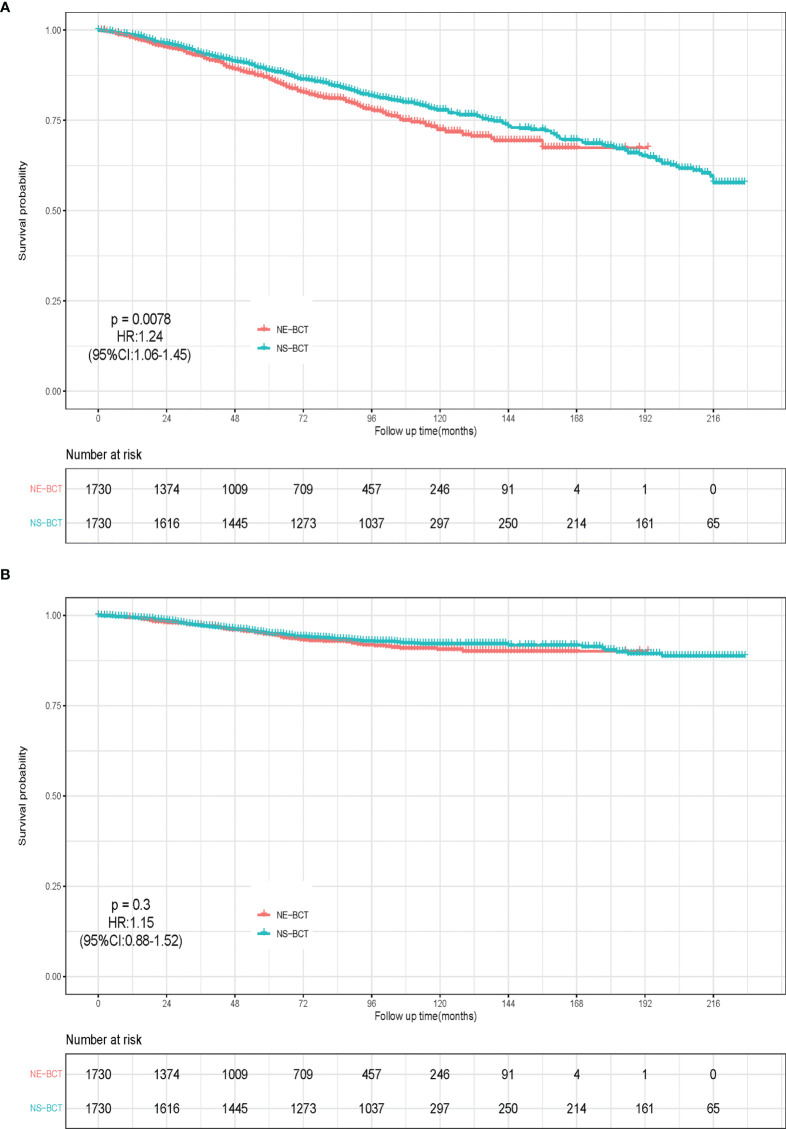
Kaplan–Meier survival analysis for NE-BCT and NS-BCT female breast cancer patients. **(A)** Overall survival curves in NE-BCT group and NS-BCT group. **(B)** Breast-cancer-specific survival curves in NE-BCT group and NS-BCT group.

After a median follow-up time of 77 months (ranging from 1 to 227 months), a total of 44,070 candidates (15.92%) died, of which 34.72% (15,300/44,070) were caused by breast cancer and 65.28% (28,770/44,070) of them died from causes unrelated to breast cancer. Kaplan–Meier analysis was employed to initially compare the effects of NE-BCT vs. NS-BCT on survival in patients with non-metastatic breast tumors after BCT. In the PSM cohort, as shown in **Figures 2A, B**, the cumulative incidences of BCSS rate at 5, 10, and 15 years were 94.75%, 92.14%, and 90.09%, respectively, in the NE-BCT group, and 94.99%, 90.59%, and 90.42%, respectively, in the NS-BCT group, while the cumulative incidences of overall survival (OS) at 5, 10, and 15 years were 86.44%, 72.44%, and 67.40%, respectively, in the NE-BCT group, and 88.86%, 77.88%, and 67.56%, respectively, in the NS-BCT group. Compared with the patients in the NS-BCT group, the cumulative incidences of BCSS at 15 years for patients in the NE-BCT group decreased by 0.33% (90.09% vs. 90.42%), while the OS incidence decreased by 0.16% (67.40% vs. 67.56%). The HRs of 1.15 (95%CI, 0.88–1.52, *p*=0.3) and 1.24 (95%CI, 1.06–1.45, *p*=0.0078) demonstrated that the administration of NE-BCT was associated with reduced OS and approximate BCSS. Additionally, similar results were found in the original cohort (**Supplementary Figures S1A, B**).

### Multivariate Cox Regression Analysis on the Factors Affecting OS

**Figure 3:**
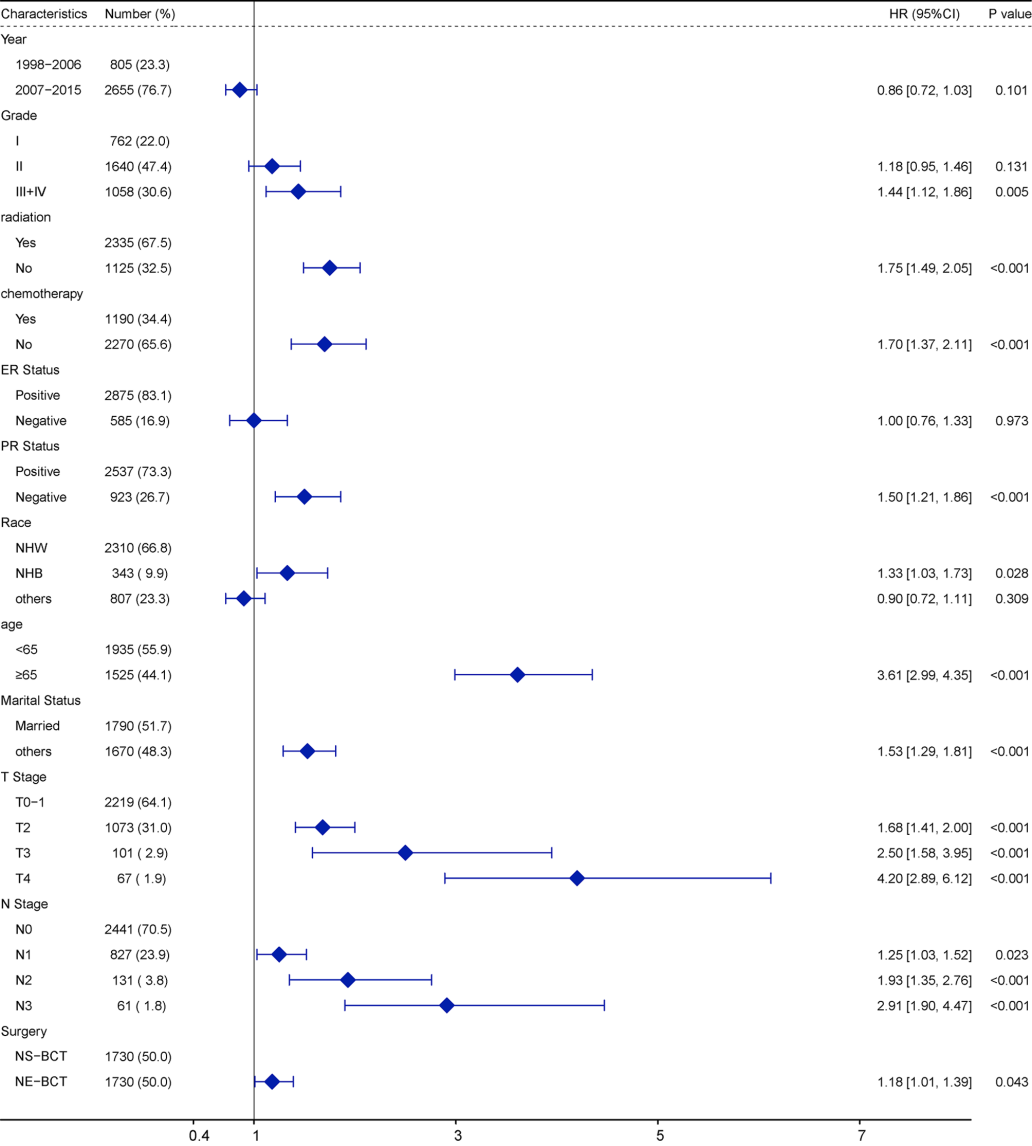
Multivariate Cox regression model forest graph.

To explore the independent prognostic factors in OS, a multivariate Cox regression model forest graph was performed (**Figure 3**). As shown in the multivariate Cox regression analysis in the PSM cohort, the clinicopathological features, such as marriage status, PR status, N stage, surgical procedures, and radiotherapy and chemotherapy status were independent prognostic factors affecting OS. The adoption of NE-BCT was associated with poorer OS (HR=1.13; 95%CI, 1.01–1.27, *p*=0.036<0.05) in comparison with NS-BCT. Similar results were archived in the original cohort (**Supplementary Figure S2**).

### Competing Risk Model Analysis

**Figure 4:**
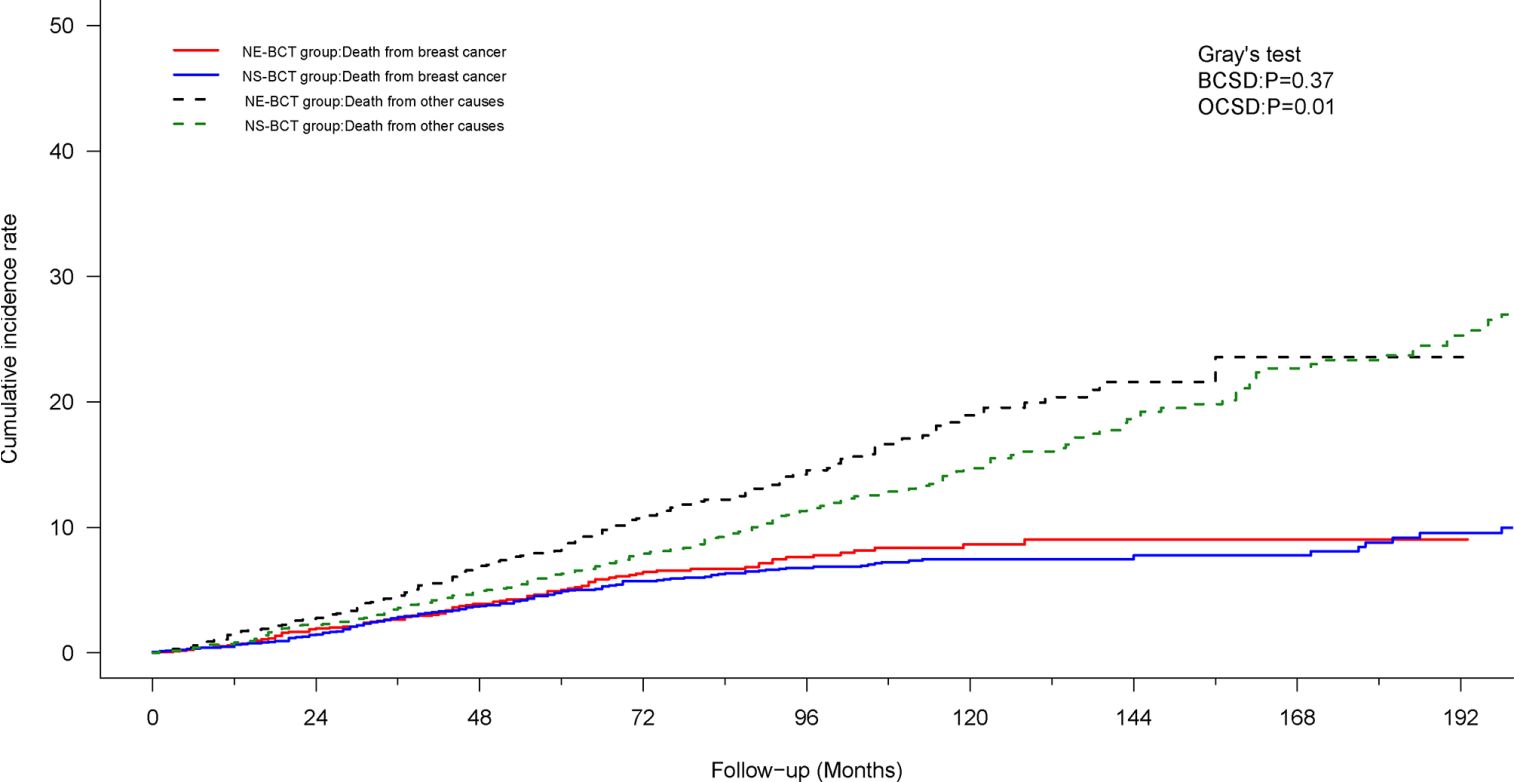
Cumulative incidence of breast-cancer-specific death (BCSD) and other causes of death (OCSD) in the NE-BCT and NS-BCT groups.

To mitigate the competing risk affecting BCSD and the occurrence of the primary events, a competing risk regression model analysis was applied. In the PSM cohort, as shown in **Figure 4**, subjects in the NE-BCT group tended to have higher cumulative OCSD incidence (Gray’s test, *p*=0.01) rather than the BCSD incidence (Gray’s test, *p*=0.37) in comparison with those in the NS-BCT group. Similar results were produced in the original cohort (**Supplementary Figure S3**).

### Multivariate Competing Risk Regression Model Analysis

To further research the independent prognostic factors affecting BCSD and OCSD, a multivariate competing risk regression model analysis was performed (**Table 2**). The administration of NE-BCT was associated with worse OCSD (HR=1.217; 95%CI, 1.002–1.478, *p*=0.048<0.05) rather than BCSD (HR=0.945; 95%CI, 0.715–1.249, *p*=0.69>0.05) in comparison with NS-BCT. The clinical pathological parameters, such as age at diagnosis, radiotherapy status, and chemotherapy status, were independent prognostic factors affecting BCSD and OCSD. The same findings were found in the original cohort (**Supplementary Table S2**).

**Table 2:** Multivariate competing risk regression model analysis.

Characteristics	BCSD (N1 = 215,32.9%)			OCSD (N2 = 438,67.1%)		
	Hazard ratio	95% CI	p-value	Hazard ratio	95% CI	p-value
Age						
<65	1	–	–	1	–	–
≧65	1.64	1.214–2.211	0.0013	5.411	4.157–7.044	<0.0001
Race						
Non-Hispanic White	1	–	–	1	–	–
Non-Hispanic Black	1.536	1.033–2.285	0.03	1.106	0.766–1.598	0.59
others	1.326	0.937–1.877	0.11	0.712	0.540–0.939	0.016
Year of diagnosis						
1998–2006	1	–	–	1	–	–
2007–2015	0.69	1.214–2.211	0.012	0.836	0.680–1.028	0.089
Marital status						
Married	1	–	–	1	–	–
others	1.25	0.940–1.675	0.12	1.61	1.310–1.977	<0.0001
Grade						
I	1	–	–	1	–	–
II	2.44	1.356–4.388	0.0029	0.99	0.787–1.244	0.93
III+IV	3.78	2.064–6.936	<0.0001	0.989	0.730–1.341	0.94
ER Status						
Positive	1	–	–	1	–	–
Negative	0.866	0.56–1.341	0.52	0.992	0.680–1.449	0.97
PR Status						
Positive	1	–	–	1	–	–
Negative	1.81	1.234–2.640	0.0024	1.206	0.931–1.562	0.16
T Stage						
T0–1	1	–	–	1	–	–
T2	2.31	1.646–3.240	<0.0001	1.256	1.017–1.552	0.034
T3	2.71	1.430–5.119	0.0022	1.445	0.618–3.377	0.4
T4	2.57	1.232–5.368	0.01	2.987	1.734–5.145	<0.0001
N Stage						
N0	1	–	–	1	–	–
N1	1.85	1.303–2.612	<0.0001	0.953	0.741–1.227	0.71
N2	3.49	2.088–5.819	<0.0001	1.025	0.566–1.858	0.93
N3	6.45	3.721–11.187	<0.0001	0.591	0.210–1.662	0.32
Surgery						
NS-BCT	1	–	–	1	–	–
NE-BCT	0.945	0.715–1.249	0.69	1.217	1.002–1.478	0.048
Radiation						
Yes	1	–	–	1	–	–
No	1.38	1.018–1.858	0.038	1.753	1.454–2.112	<0.0001
Chemotherapy						
Yes	1	–	–	1	–	–
No	1.04	0.731–1.481	0.83	2.311	1.681–3.178	<0.0001

NE-BCT, nipple-excising breast-conserving therapy; NS-BCT, nipple-sparing breast-conserving therapy; ER, estrogen receptor; PR, progesterone receptor; HER-2, human epidermal growth factor receptor 2; BCSD, breast-cancer-specific death; OCSD, other-cause-specific death.

### Subgroup Analysis of Patients Between 2010 and 2015 With Known HER-2 Status

**Figure 5:**
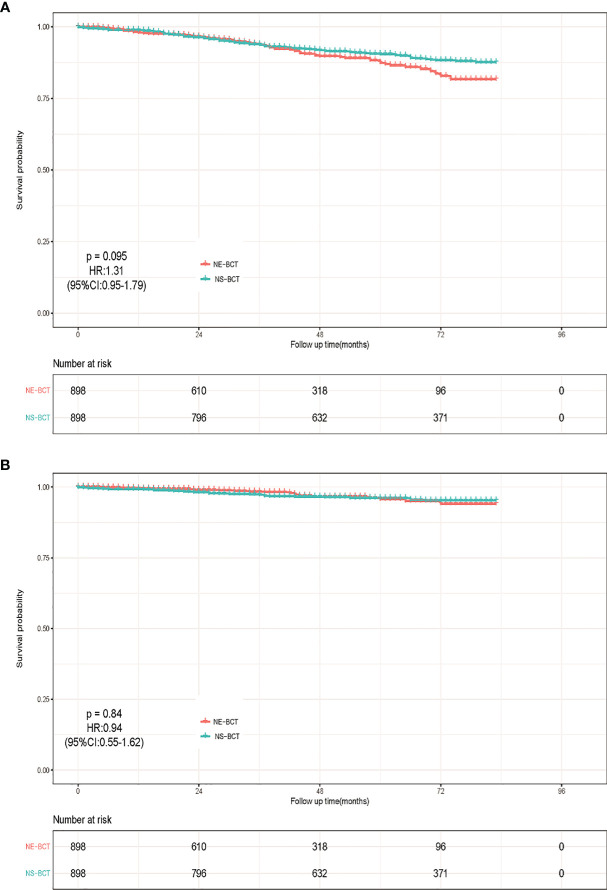
Kaplan–Meier survival analysis for NE-BCT and NS-BCT female breast cancer patients between 2010 and 2015. **(A)** Overall survival curves in the NE-BCT and NS-BCT groups. **(B)** Breast-cancer-specific survival curves in the NE-BCT and NS-BCT groups.

**Figure 6:**
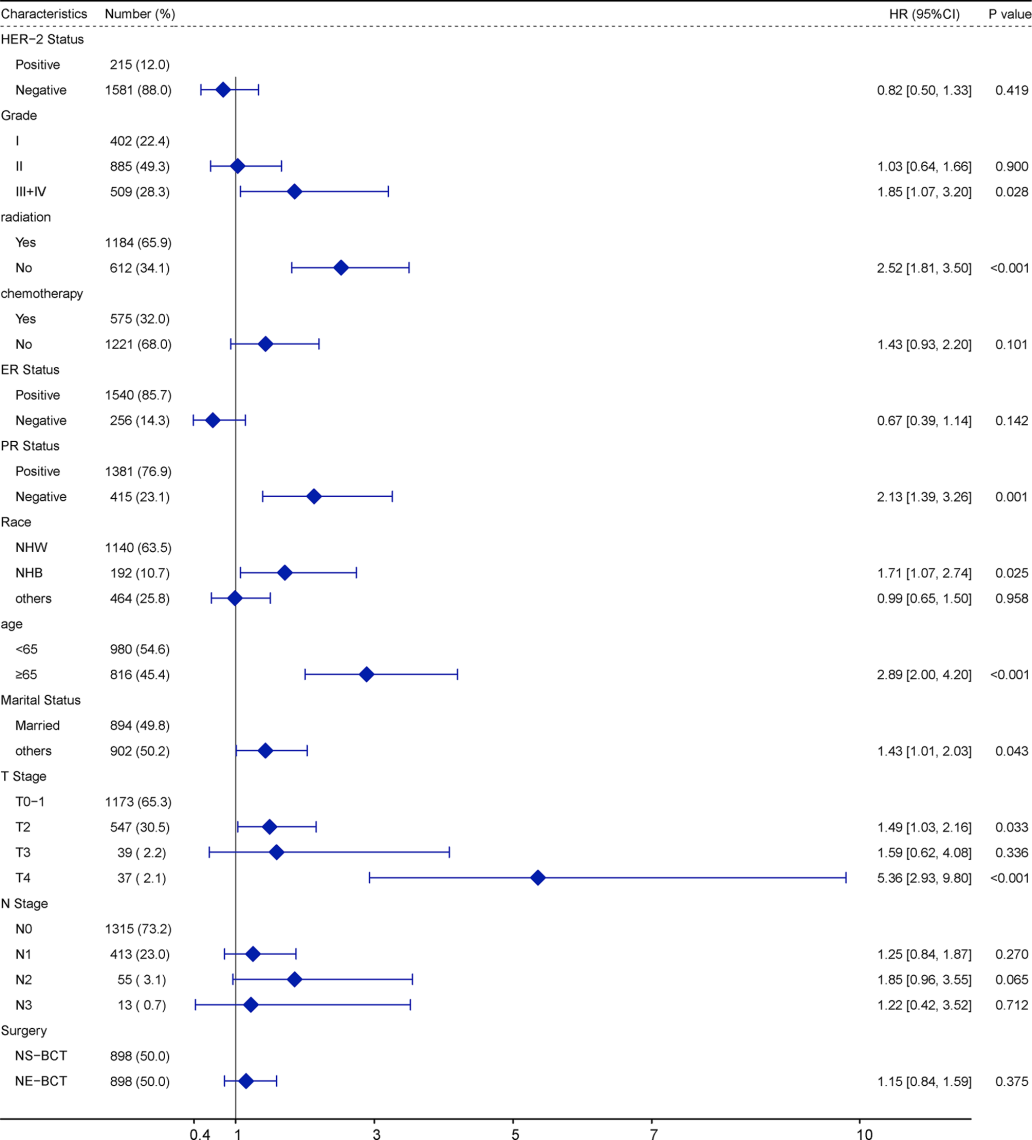
Multivariate Cox regression model forest graph between 2010 and 2015.

**Figure 7:**
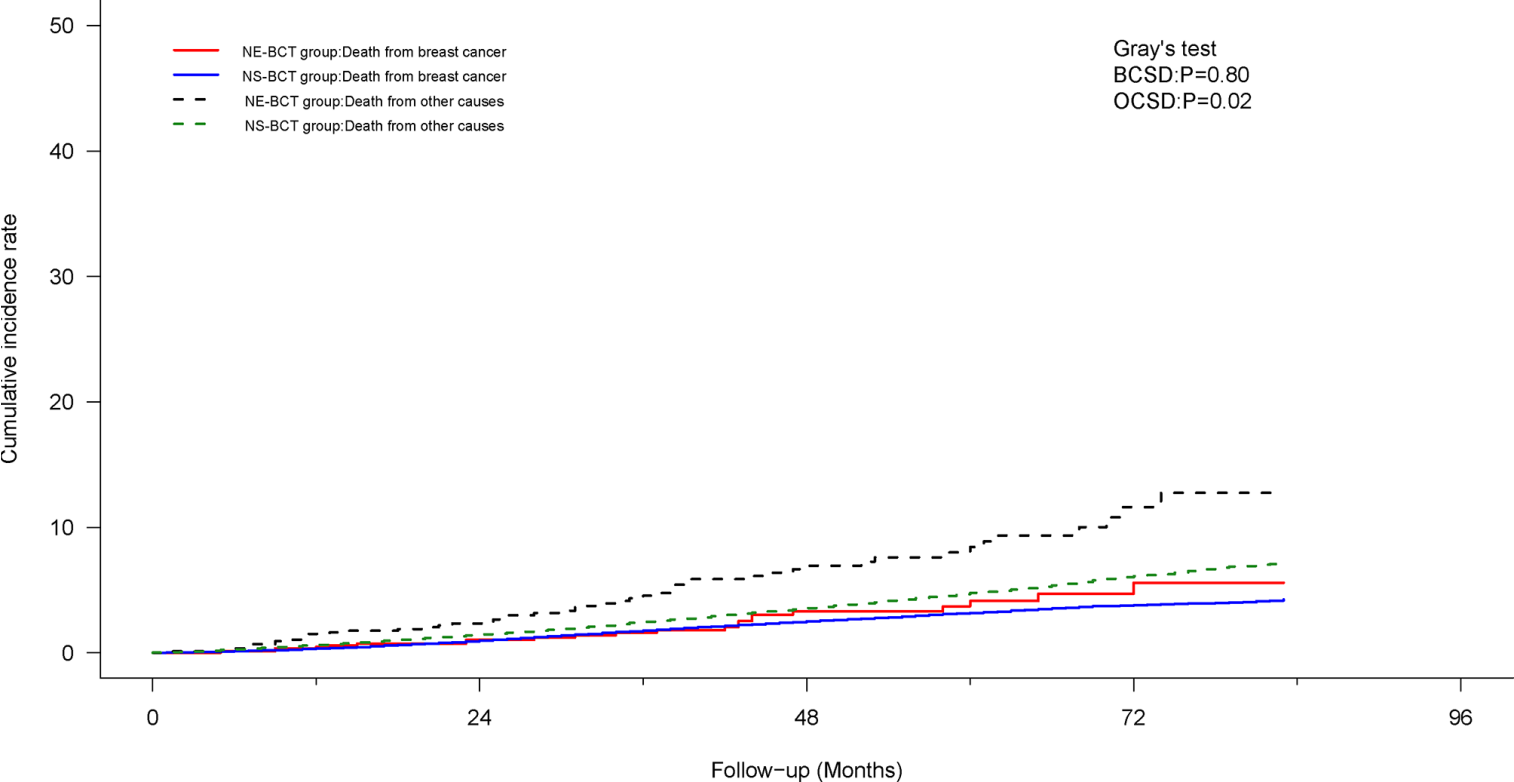
Cumulative incidence of breast-cancer-specific death (BCSD) and other causes of death (OCSD) in the NE-BCT and NS-BCT groups between 2010 and 2015.

Moreover, to investigate the prognostic value of human epidermal growth factor receptor 2 (HER-2) status based on NE-BCT vs. NS-BCT, a subgroup analysis based on patients diagnosed between 2010 and 2015 was performed (**Table 3**). As shown in **Figures 5A, B**, unexpectedly, patients in the NE-BCT group tended to have similar OS (HR=1.31; 95%CI, 0.95–1.79, *p*=0.095>0.05) and BCSS (HR=0.94; 95%CI, 0.55–1.62, *p*=0.84>0.05) to those in the NS-BCT group. After multivariate Cox regression and competing risk regression model analyses, neither surgery methods nor HER-2 status was an independent prognostic factor affecting OS (**Figure 6**) or BCSD (**Figure 7**; **Table 4**). The same findings were found in the original cohort (**Supplementary Figures S4**–**S6** **;** **Supplementary Tables S3**, **S4**).

**Table 3:** Patient clinical and pathological characteristics between 2010 and 2015.

Characteristics	All patients	NE-BCT	NS-BCT	χ2/F	p-value
		N = 1,796	N = 898	N = 898	
Age at diagnosis (median)	62.70 ± 12.94	62.58 ± 12.96	62.81 ± 12.95	0.708	>0.05
Age				0.036	0.85
<65	980 (54.6)	492 (54.8)	488 (54.3)		
≧65	816 (45.4)	406 (45.2)	410 (45.7)		
Race				0.155	0.926
Non-Hispanic White	1140 (63.5)	574 (63.9)	566 (53.0)		
Non-Hispanic Black	192 (10.7)	95 (10.6)	97 (10.8)		
Others	464 (25.8)	229 (25.5)	235 (23.2)		
Marital status				0.036	0.85
Married	894 (49.8)	449 (50.0)	445 (49.6)		
Others	902 (50.2)	449 (50.0)	453 (50.4)		
Laterality				0.009	0.925
Left	970 (54.0)	484 (53.9)	486 (54.1)		
Right	826 (46.0)	414 (46.1)	412 (45.9)		
Grade				0.028	0.986
I	402 (22.4)	201 (22.4)	201 (22.4)		
II	885 (49.3)	441 (49.1)	444 (49.4)		
III+IV	509 (28.3)	256 (28.5)	253 (28.2)		
ER Status				0.073	0.087
Positive	1540 (85.7)	768 (85.5)	772 (86.0)		
Negative	256 (14.3)	130 (14.5)	126 (14.0)		
PR Status				0.003	0.955
Positive	1385 (77.1)	690 (76.8)	691 (76.9)		
Negative	415 (22.9)	208 (23.2)	207 (23.1)		
HER2 Status				0.639	0.424
Positive	215 (12.0)	113 (12.6)	102 (11.4)		
Negative	1581 (88.0)	785 (87.4)	796 (88.6)		
T Stage				1.538	0.646
T0–1	1173 (65.3)	587 (65.4)	586 (65.3)		
T2	547 (30.5)	268 (29.8)	279 (31.1)		
T3–4	76 (4.2)	43 (4.8)	33 (3.7)		
N Stage				0.068	0.967
N0	1,315 (73.2)	659 (73.4)	656 (73.0)		
N1	413 (23.0)	206 (22.9)	207 (23.1)		
N2–3	68 (3.8)	33 (3.7)	35 (3.9)		
Radiation				0.01	0.921
Yes	1,184 (65.9)	593 (66.0)	591 (65.8)		
No	612 (34.1)	305 (34.0)	307 (34.2)		
Chemotherapy				0.003	0.96
Yes	575 (32.0)	287 (32.0)	288 (32.1)		
No	1,221 (68.0)	611 (68.0)	610 (67.9)		

NE-BCT, nipple-excising breast-conserving therapy; NS-BCT, nipple-sparing breast-conserving therapy; ER, estrogen receptor; PR, progesterone receptor; HER-2, human epidermal growth factor receptor 2; BCSD, breast-cancer-specific death; OCSD, other-cause-specific death.

**Table 4:** Multivariate competing risk regression model analysis for patients between 2010 and 2015.

Characteristics	BCSD(N1 = 57,35.4%)			OCSD(N2 = 104,64.6%)		
	Hazard ratio	95% CI	p-value	Hazard ratio	95% CI	p-value
Age						
<65	1	–	–	1	–	–
≧65	2.498	1.4–4.46	0.0019	2.998	1.8699–1.806	<0.0001
Race						
Non-Hispanic White	1	–	–	1	–	–
Non-Hispanic Black	1.392	0.648–2.99	0.4	1.743	0.937–3.244	0.079
others	1.676	0.893–3.14	0.11	0.74	0.4108–1.334	0.32
Marital status						
Married	1	–	–	1	–	–
others	1.348	0.74–2.46	0.33	1.457	0.9377–2.264	0.094
Grade						
I	1	–	–	1	–	–
II	5.902	0.803–43.29	0.081	0.845	0.5087–1.402	0.51
III+IV	13.865	1.88–102.24	0.0099	1.028	0.4972–2.123	0.94
ER Status						
Positive	1	–	–	1	–	–
Negative	0.694	0.303–1.59	0.39	0.775	0.3632–1.653	0.51
PR Status						
Positive	1	–	–	1	–	–
Negative	2.248	1.158–4.36	0.017	1.718	1.0177–2.899	0.043
HER2 Status						
Positive	1	–	–	1	–	–
Negative	1.222	0.525–2.85	0.64	0.543	0.2956–0.999	0.05
T Stage						
T0–1	1	–	–	1	–	–
T2	2.15	1.126–4.11	0.02	1.147	0.6979–1.886	0.59
T3	2.168	0.557–8.43	0.26	1.178	0.3187–4.352	0.81
T4	2.157	0.617–7.55	0.23	5.745	2.5787–12.798	<0.0001
N Stage						
N0	1	–	–	1	–	–
N1	1.687	0.842–3.38	0.14	1.151	0.6686–1.982	0.61
N2	4.012	1.549–10.39	0.0042	0.539	0.1192–2.436	0.42
N3	2.869	0.785–10.48	0.11	0.646	0.0756–5.518	0.69
Surgery						
NS-BCT	1	–	–	1	–	–
NE-BCT	0.821	0.467–1.45	0.49	1.472	0.9829–2.204	0.061
Radiation						
Yes	1	–	–	1	–	–
No	2.242	1.249–4.03	0.0069	2.419	1.5985–3.659	<0.0001
Chemotherapy						
Yes	1	–	–	1	–	–
No	0.738	0.363–1.5	0.4	2.789	1.5004–5.186	0.0012

NE-BCT, nipple-excising breast-conserving therapy; NS-BCT, nipple-sparing breast-conserving therapy; ER, estrogen receptor; PR, progesterone receptor; HER-2, human epidermal growth factor receptor 2; BCSD, breast-cancer-specific death; OCSD, other-cause-specific death.

## Discussion

In this retrospective study, based on a large cohort of 276,661 candidates in the SEER database from 1998 to 2015 using PSM analysis, we found that subjects in the NE-BCT group had similar BCSS to those in the NS-BCT group. To our knowledge, this was the first and largest statistical study based on a large population to directly assess the efficiency of NE-BCT on patients with non-metastatic breast carcinoma through analyzing the demographic and pathological features and survival variables.

BCT followed by whole breast radiotherapy has been established as a good treatment option for breast cancer for several decades (3, 4). The nipple–areola complex (NAC) is a signature of the breast, and the preservation of breast and NAC in combination of postoperative radiotherapy has emerged as a standard treatment for those with early stage disease. The preservation of NAC means a positive impact on patients’ satisfaction with cosmetic results and feeling of mutilation among women (5–7, 13, 19, 20). It is predictable that the excision of NAC has an adverse effect on quality of life and the consequent long-term survival. This speculation about the role of NE-BCT is confirmed in our study. After Kaplan–Meier curve analysis, candidates in the NE-BCT group had poorer OS than those in the NS-BCT group. However, the impact resulting from the differences in the clinicopathological features, such as age at diagnosis, marital status, degree of histological differentiation, T and N stage, and radiotherapy and chemotherapy status, could not be neglected.

Clinicopathological characteristics, such as degree of histological differentiation and TNM stage, as reported previously (21, 22), were closely correlated with the long-term prognosis in patients with FBC. In our study, a disparity in the patients enrolled, which could contribute to selection bias, was found in nearly all key methodological characteristics in the original cohort. As a consequence, a propensity score matching analysis was employed to balance the differences in clinicopathological factors in our study. As expected, the differences among the clinical and pathological characteristics no longer appeared in the PSM cohort. Therefore, the findings in the PSM cohort could make the study to more objectively maximize reflection of the differences between the two groups. After PSM analysis, subjects in the NE-BCT group still tended to have more unfavorable OS than those in the NS-BCT group through Kaplan–Meier curve analysis. Such result indicated that the administration of NE-BCT had adverse effects in women with early FBC. However, the potential misinterpreting factors, which might preclude the occurrence of the primary event, should not be disregarded.

To estimate and eliminate the confounding factors that had been recognized as valuable prognostic indexes in OS for breast cancer patients, a multivariable Cox regression analysis was employed, with the HRs and corresponding 95% CIs performed. Then, we found that NE-BCT was an independent debilitating factor affecting OS, whether in the PSM cohort or the original cohort. In addition, age of 65 years and above at diagnosis, unmarried status, PR-negative status, and no radiotherapy or chemotherapy were adversely independent prognosis factors for patients with BCT. These results were similar to those of other studies (3, 4, 23–25). However, the estimation bias resulting from OCSD, which might be a competing risk affecting BCSD and preclude the occurrence of the primary event, should not be overlooked (26–28).

To undermine the underlying estimation bias and further investigate the efficacy of NE-BCT on BCSD or OCSD, the Fine and Gray competing risk model and multivariable competing risk regression analysis were carried out. Then, we found that subjects in the NE-BCT group tended to have higher cumulative OCSD rather than BCSD when compared with those in the NS-BCT group. The underlying reason could be that the inexistence of NAC, which meant imperfect cosmetic outcomes or psychosocial and sexual wellbeing (3, 4), might affect the incidence of OCSD in patients with breast cancer and then increased the cumulative incidence of OCSD. Consequently, the existence of NAC plays a vital part in decreasing OCSD rather than BCSD and could be a secure and effective technique in surgical treatment regimen for early FBC patients.

Breast cancer with the amplification of the HER2 gene and/or overexpression of HER2, which are known to be more clinically aggressive with poorer long-term prognosis, represents 11%–30% of all breast tumor (29). To further explore the predictive consequences of NE-BCT vs. NS-BCT on breast cancer based on HER2 status, a subgroup analysis was performed. Through multivariate Cox regression and Fine–Gray multivariable regression analyses in the PSM cohort, unexpectedly, patients in the NE-BCT and NS-BCT group tended to have similar OS and BCSS in spite of HER2 status. Additionally, HER-2 status was only borderline corrected with OCSD rather than BCSD in our study. Such results were consistent with that of previous studies (30, 31) that reported that HER2-enriched tumor was not an independent prognostic predictor for patients with BCT. However, we still need a larger cohort study to elaborate the effect of NE-BCT vs. NS-BCT on breast cancer based on HER2 status for patients with BCT.

This study also has limitations. First, due to the nature of the retrospective analysis, selection and confounding biases could not be ignored. A randomized controlled trial that randomly assigns patients into different groups by treatment methods should be designed. Second, the detailed sequence and regimens between BCT and chemotherapy, targeted therapy against HER-2/neu-overexpression, and endocrine therapy, radiation dose, and clinical target volume were unavailable in our study. Information regarding the status of locoregional and distant recurrence after comprehensive treatment also could not be obtained. Third, due to the detailed operation procedures of the patients unobtainable in our study, the unrevealed indications for NE-BCT will affect the accuracy of the study. Finally, and most importantly, median follow-up time was not long enough in the HER-2 status subgroup analysis. Longer follow-up time is necessary for an accurate assessment of prognostic factors for patients. However, we suggest that the findings of this study, which covers about 28% of the US population of patients with cancer, are generalizable and will contribute to improved survival in non-metastatic patients.

## Conclusion

Our study demonstrated that NE-BCT is one oncologically safe and effective surgical treatment and can be widely recommended in clinics for women with non-metastatic breast cancer. Randomized controlled clinical trials with longer follow-up time are still needed to provide a high level of evidence on advantages of NE-BCT for patients with non-metastatic metaplastic FBC patients.

## Data Availability Statement

The original contributions presented in the study are included in the article/**Supplementary Material**. Further inquiries can be directed to the corresponding author.

## Ethics Statement

Written informed consent was not obtained from the individual(s) for the publication of any potentially identifiable images or data included in this article.

## Author Contributions

SL and YZha drafted the manuscript and analyzed data. ZY, LY, and YZho generated the figure. PQ performed the background research. CZ and JH edited the manuscript. All authors contributed to the article and approved the submitted version.

## Funding

This work was supported by the National Natural Science Foundation of China (Grant No. 81502413) and Shaanxi Provincial Natural Science Foundation of China (Grant No. 2019SF-145).

## Conflict of Interest

The authors declare that the research was conducted in the absence of any commercial or financial relationships that could be construed as a potential conflict of interest.

## Publisher’s Note

All claims expressed in this article are solely those of the authors and do not necessarily represent those of their affiliated organizations, or those of the publisher, the editors and the reviewers. Any product that may be evaluated in this article, or claim that may be made by its manufacturer, is not guaranteed or endorsed by the publisher.

## Supplementary Material

Supplementary Figure 1 Kaplan–Meier survival analysis for NE-BCT and NS-BCT female breast cancer patients in the original cohort. **(A)** Overall survival curves in NE-BCT group and NS-BCT group. **(B)** Breast cancer specific survival curves in NE-BCT group and NS-BCT group.

Supplementary Figure 2 Multivariate Cox regression model forest graph in the original cohort.

Supplementary Figure 3 Cumulative incidence of breast cancer-specific of death (BCSD) and other causes of death (OCSD) in NE-BCT group and NS-BCT group in the original cohort.

Supplementary Figure 4 Kaplan–Meier survival analysis for NE-BCT and NS-BCT female breast cancer patients between 2010 and 2015 before PSM. **(A)** Overall survival curves in NE-BCT group and NS-BCT group. **(B)** Breast cancer specific survival curves in NE-BCT group and NS-BCT group.

Supplementary Figure 5 Multivariate Cox regression model forest graph between 2010 and 2015 before PSM.

Supplementary Figure 6 Cumulative incidence of breast cancer-specific of death (BCSD) and other causes of death (OCSD) in NE-BCT group and NS-BCT group between 2010 and 2015 before PSM.

## Abbreviations

BCT, breast-conserving therapy; NE-BCT, nipple-excising breast-conserving therapy; NS-BCT, nipple-sparing breast-conserving therapy; NAC, nipple areolar complex; DCIS, ductal carcinoma *in situ*; FBC, female breast cancer; BCSS, breast-cancer-specific survival; OS, overall survival; BCSD, breast-cancer-specific death; OCSD, other-causes-specific death; ER, estrogen receptor; PR, progesterone receptor; HER-2, human epidermal growth factor receptor 2.
