# Supplementary figures and images for: Effect of the Nipple-Excising Breast-Conserving Therapy in Female Breast Cancer: A Competing Risk Analysis and Propensity Score Matching Analysis of Results Based on the SEER Database
Source: Front Oncol. 2022 Apr 14;12:848187. doi: 10.3389/fonc.2022.848187 (PMC9048049; doi:10.3389/fonc.2022.848187)

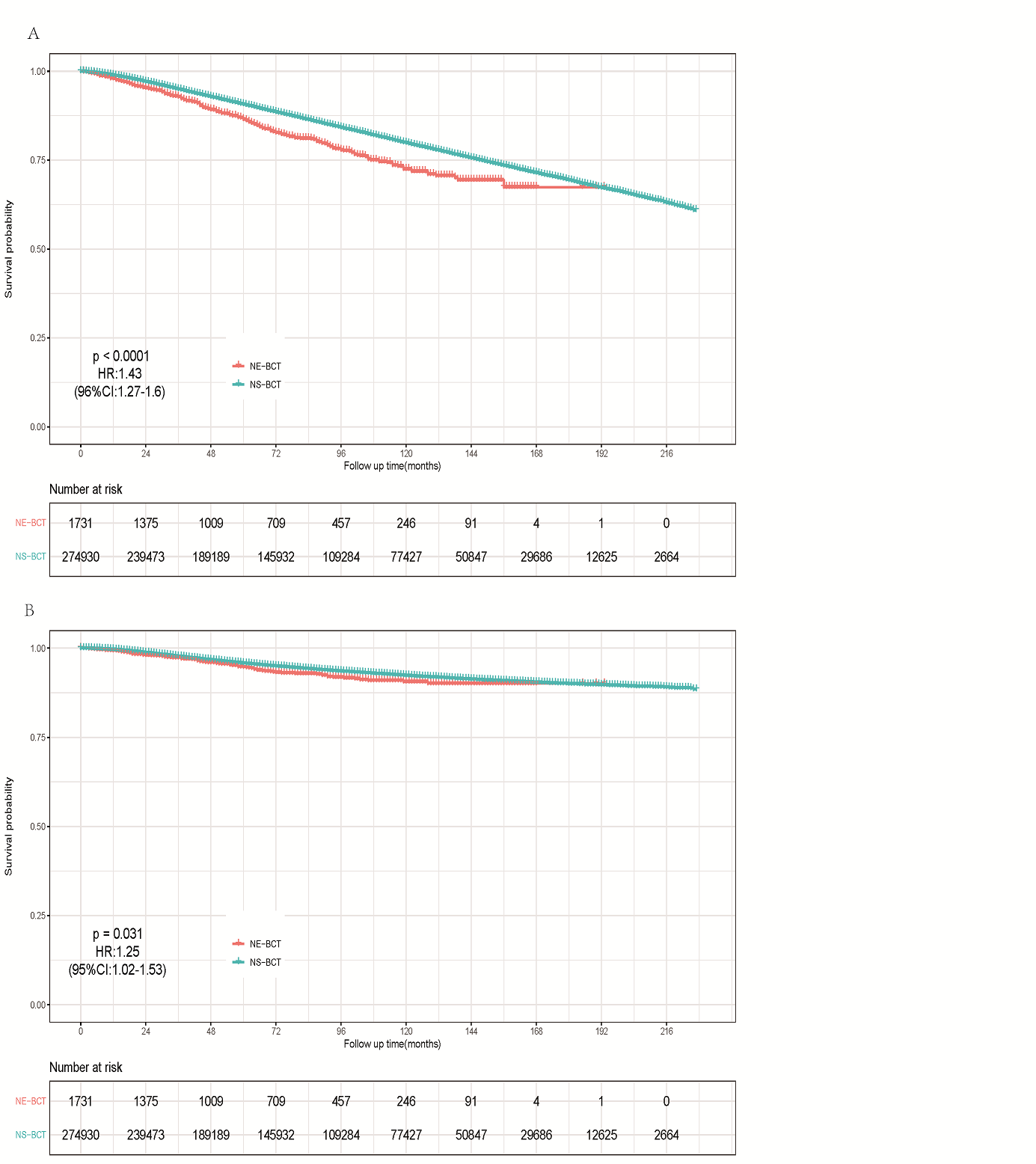

Supplement: Supplementary Figure 1 — Kaplan–Meier survival analysis for NE-BCT and NS-BCT female breast cancer patients in the original cohort. (A) Overall survival curves in NE-BCT group and NS-BCT group. (B) Breast cancer specific survival curves in NE-BCT group and NS-BCT group. [file Image_1.tif]

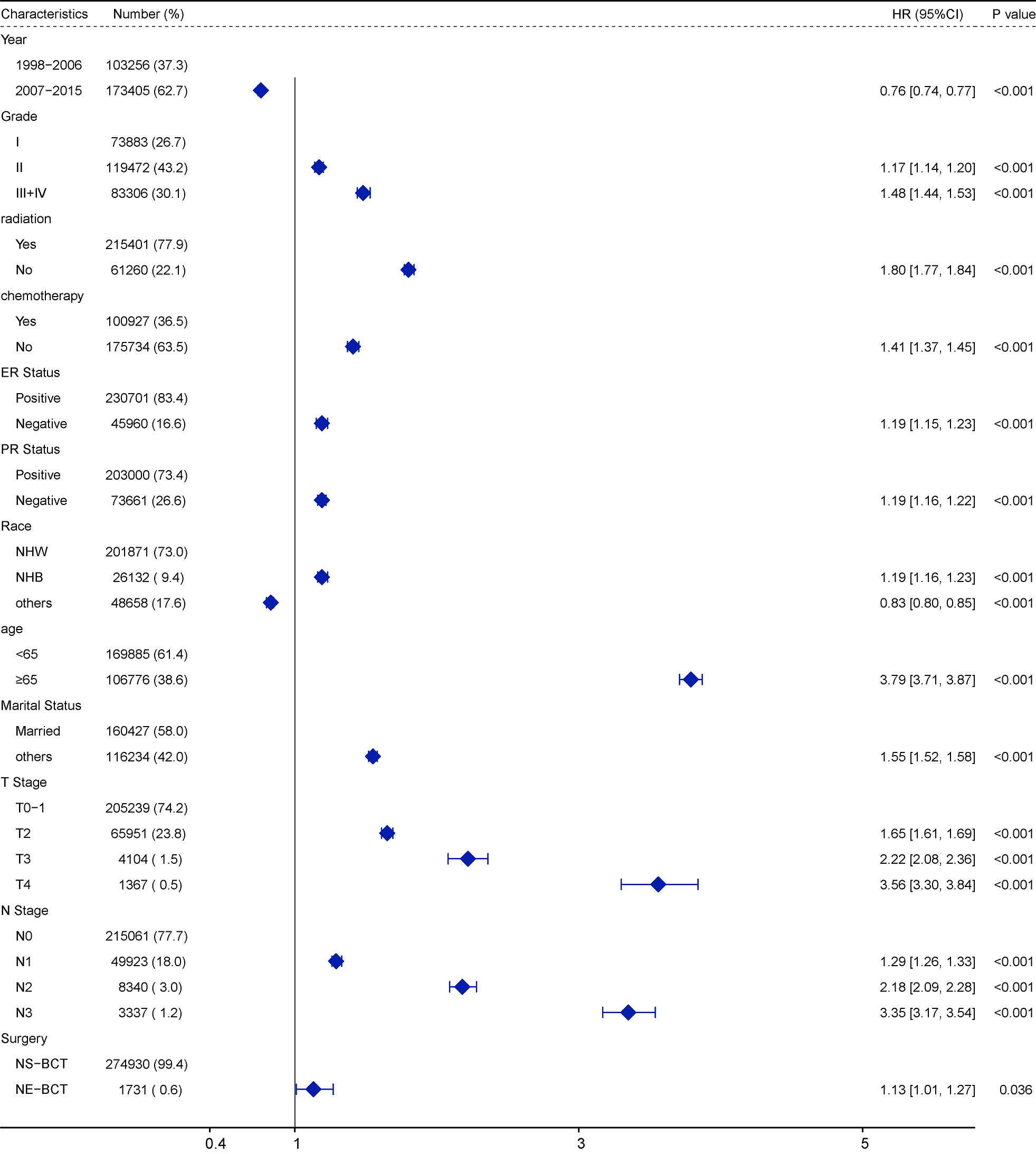

Supplement: Supplementary Figure 2 — Multivariate Cox regression model forest graph in the original cohort. [file Image_2.tif]

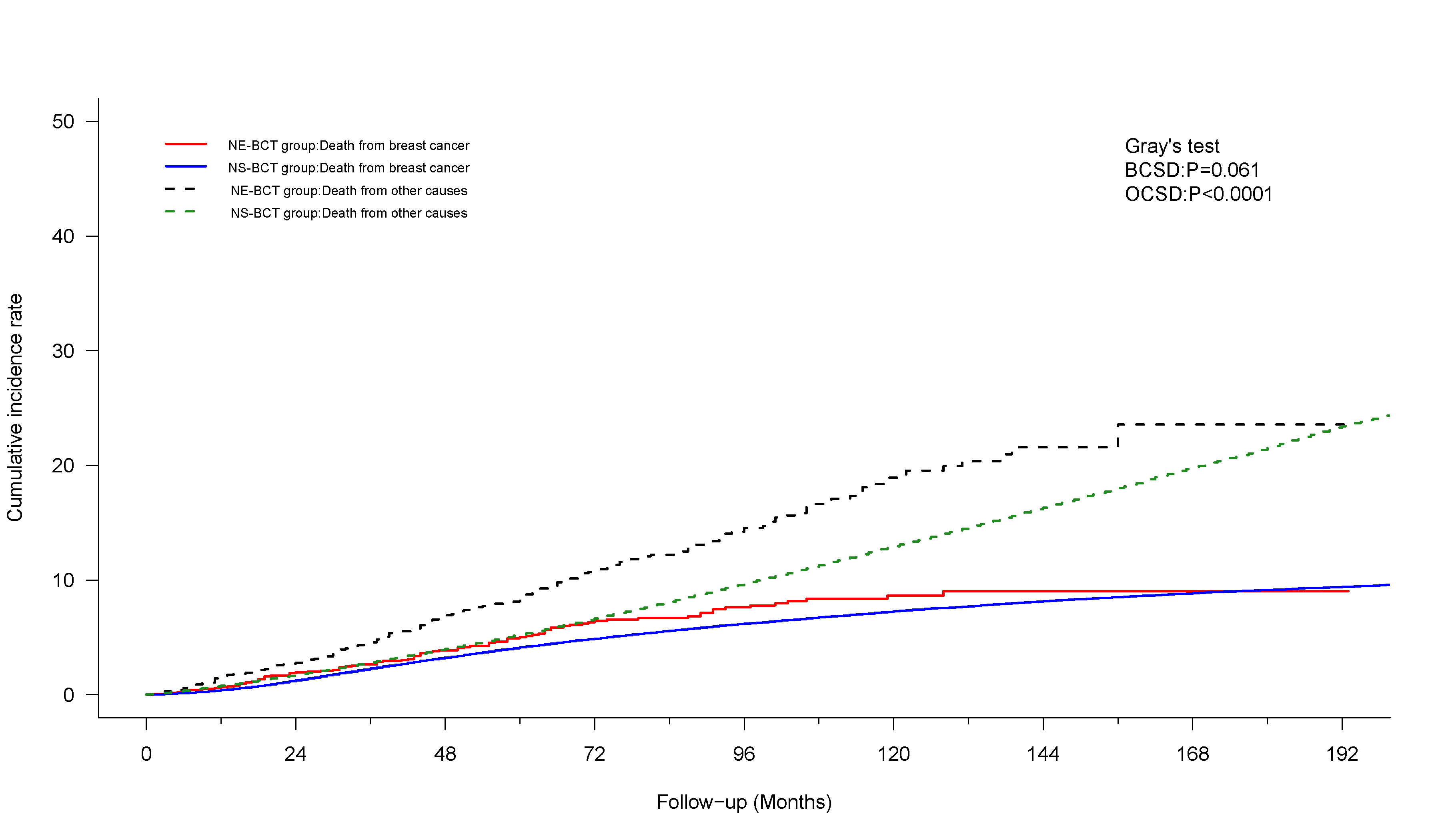

Supplement: Supplementary Figure 3 — Cumulative incidence of breast cancer-specific of death (BCSD) and other causes of death (OCSD) in NE-BCT group and NS-BCT group in the original cohort. [file Image_3.tif]

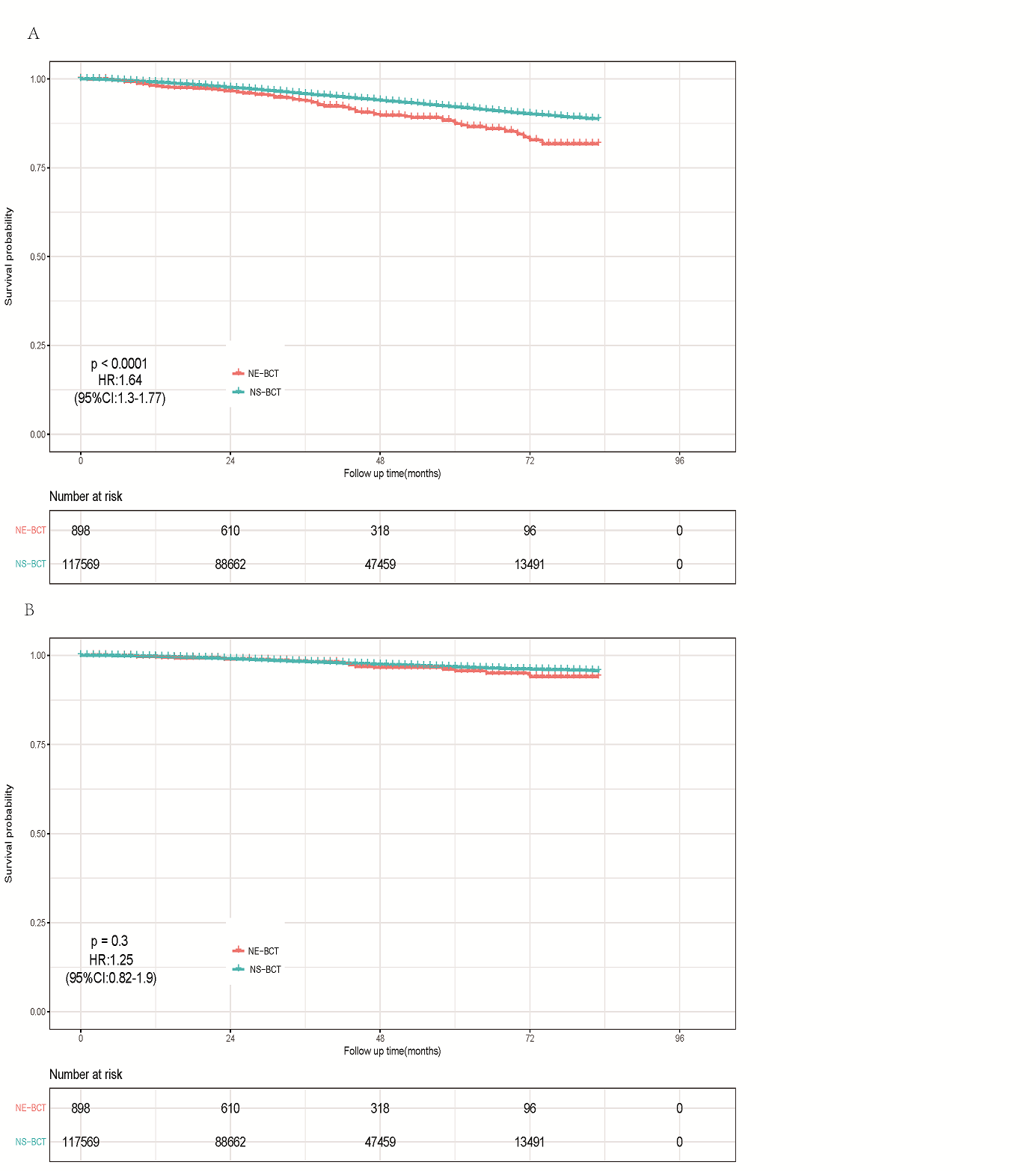

Supplement: Supplementary Figure 4 — Kaplan–Meier survival analysis for NE-BCT and NS-BCT female breast cancer patients between 2010 and 2015 before PSM. (A) Overall survival curves in NE-BCT group and NS-BCT group. (B) Breast cancer specific survival curves in NE-BCT group and NS-BCT group. [file Image_4.tif]

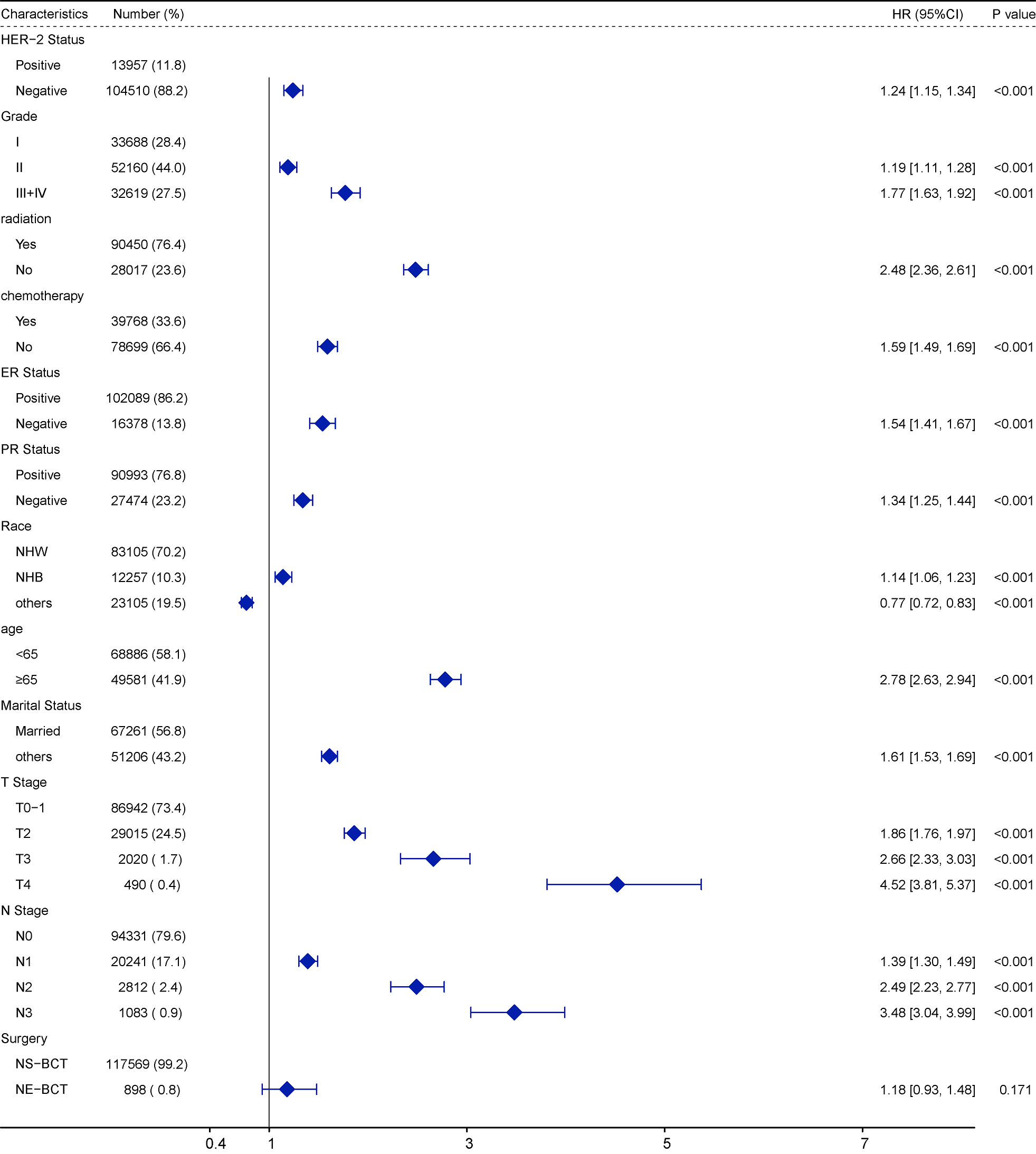

Supplement: Supplementary Figure 5 — Multivariate Cox regression model forest graph between 2010 and 2015 before PSM. [file Image_5.tif]

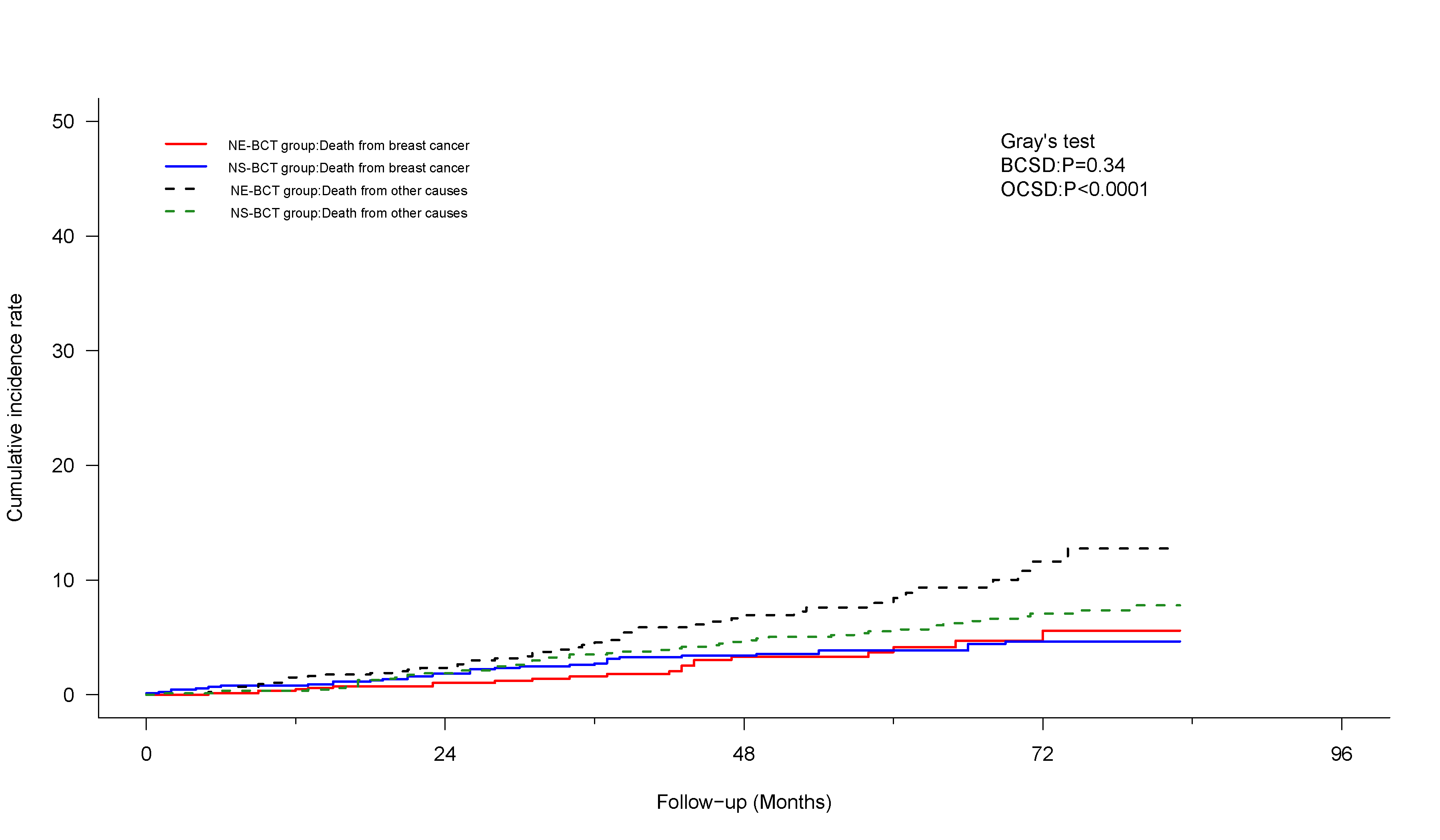

Supplement: Supplementary Figure 6 — Cumulative incidence of breast cancer-specific of death (BCSD) and other causes of death (OCSD) in NE-BCT group and NS-BCT group between 2010 and 2015 before PSM. [file Image_6.tif]
